# The Association Between Genetic Polymorphisms of *UGT1A1*, *ABCG2*, and *NR1I2* and Dolutegravir Pharmacokinetic Parameters in Thai People Living with HIV

**DOI:** 10.3390/pharmaceutics17111499

**Published:** 2025-11-20

**Authors:** Anan Chanruang, Angela K. Birnbaum, Sasithorn Sirilun, Suthunya Chupradit, Sasiwimol Ubolyam, Napon Hiranburana, Yong Soon Cho, Jae Gook Shin, Anchalee Avihingsanon, Baralee Punyawudho

**Affiliations:** 1 PhD Degree Program in Pharmacy, Faculty of Pharmacy, Chiang Mai University, Chiang Mai 50200, Thailand; anan_c@cmu.ac.th; 2 Department of Experimental and Clinical Pharmacology, College of Pharmacy, University of Minnesota, Minneapolis, MN 55455, USA; birnb002@umn.edu; 3 Department of Pharmaceutical Sciences, Faculty of Pharmacy, Chiang Mai University, Chiang Mai 50200, Thailand; sasithorn.s@cmu.ac.th; 4 Department of Pharmaceutical Care, Faculty of Pharmacy, Chiang Mai University, Chiang Mai 50200, Thailand; suthunya.chu@cmu.ac.th; 5 The HIV Netherlands Australia Thailand Research Collaboration (HIV-NAT), Thai Red Cross AIDS Research and Infectious Diseases Centre, Bangkok 10330, Thailand; 6 Center for Personalized Precision Medicine of Tuberculosis, Inje University College of Medicine, Busan 47392, Republic of Korea; 7 Center of Excellence in Tuberculosis, Faculty of Medicine, Chulalongkorn University, Bangkok 10330, Thailand

**Keywords:** dolutegravir, people living with HIV, anti-retroviral medication, pharmacokinetics, genetic polymorphisms, *UGT1A1*, *ABCG2*, *NR1I2*

## Abstract

**Background/Objectives**: Dolutegravir (DTG) is recommended as first-line treatment for Thai people living with HIV (PLWH). Real-world studies show high plasma concentration variability, which may increase neuropsychiatric adverse effects. This variability can be influenced by both genetic and nongenetic factors, but data for the Thai population are insufficient. We investigated factors associated with DTG pharmacokinetics in Thai PLWH. **Methods**: A cross-sectional analysis was conducted in Thai PLWH receiving a 50 mg DTG-based regimen. Intensive blood sampling was performed to determine DTG pharmacokinetic parameters using a non-compartmental analysis. Genotyping for *UGT1A1*, *ABCG2*, and *NR1I2* was performed. Univariable and multivariable linear regression analyses were used to identify factors associated with DTG pharmacokinetics. **Results**: A total of 104 Thai PLWH were included. Multivariable analysis demonstrated that both the *UGT1A1* poor metabolizer phenotype and body weight were independently associated with DTG exposure. After adjusting for body weight, the *UGT1A1* poor metabolizer phenotype was associated with increases of 5.18% in AUC_0–24_ and 20.59% in C_trough_. No significant association was found between the *ABCG2* 421 C>A polymorphism and DTG pharmacokinetic parameters. **Conclusions**: Body weight and the *UGT1A1* poor metabolizer phenotype significantly impacted DTG exposure in Thai PLWH. Those with the *UGT1A1* poor metabolizer, particularly with lower body weight, had significantly increased DTG exposures. These findings highlight that dose optimization may be worth exploring in selected individuals in this population.

## 1. Introduction

Dolutegravir (DTG), a second-generation integrase strand inhibitor (INSTI), is recommended as first-line treatment for people living with HIV (PLWH), including adults, children, and pregnant women [1,2]. The 2021/2022 Thailand National guidelines for HIV/AIDS diagnosis, treatment, and prevention recommend a fixed-dose combination of a DTG-based regimen, comprising DTG and tenofovir alafenamide (TAF) or tenofovir disoproxil fumarate (TDF) plus lamivudine (3TC) or emtricitabine (FTC) [2]. DTG exhibits high potency, with a once-daily dosage of 50 mg yielding trough concentrations (C_trough_) that are 13 times higher than the in vitro protein-adjusted 90% inhibitory concentration (IC90) of 0.064 mg/L [3]. Although once-daily DTG has demonstrated superior efficacy and potency for viral suppression [4,5], concerns have arisen regarding neuropsychiatric adverse events (NP-AEs), with evidence suggesting that higher DTG concentrations may increase the risk of NP-AEs. This could potentially lead to treatment discontinuation [6,7,8,9]. Optimizing DTG dosing is therefore important for maximizing efficacy and minimizing toxicity. Pre-licensing trials indicated moderate pharmacokinetic variability for DTG, with coefficients of variation (%CV) ranging from 24% to 26% [3]. However, real-world studies found this variability is substantially greater (%CV up to 85%) [10]. Both non-genetic factors (e.g., sex, age, body weight, and total bilirubin) and genetic factors contribute to this variability [11,12,13,14,15,16,17], making it difficult to fully predict individual DTG exposure.

DTG is predominantly metabolized by uridine diphosphate glucuronosyltransferase family 1 member A1 (UGT1A1) and to a lesser extent by cytochrome P450 (CYP) 3A4 [18]. UGT1A1 is a member of the Phase II enzyme and is responsible for the glucuronidation of a wide range of compounds. The wild-type allele designated as UGT1A1*1 comprises six repeats (TA6) and has normal enzyme activity [19]. Five polymorphic variants are of clinical significance to UGT1A1 activity (UGT1A1*6, UGT1A1*27, UGT1A1*28, UGT1A1*36, and UGT1A1*37); three of these variants influence the tandem repeat of the TATA box ((TA5)—UGT1A1*36, (TA7)—UGT1A1*28, and (TA8)—UGT1A1*37) [19]. The polymorphisms of the *UGT1A1* gene that lead to reduced enzyme activity (e.g., *28 and *37 alleles) have been demonstrated to influence the pharmacokinetics of cabotegravir and raltegravir [20,21,22]. Thus, it is possible that DTG exposure may be influenced by *UGT1A1* polymorphisms. Prior studies reported higher DTG exposures in PLWH carrying UGT1A1*28 and the homozygous variant of UGT1A1*6 [10,23]. Additionally, evidence found individuals carrying the UGT1A1*6, UGT1A1*28, or both alleles exhibited a higher cumulative incidence of selected NP-AEs compared to individuals with the normal alleles [23]. DTG is also a substrate for breast cancer resistance protein (BCRP), encoded by the *ABCG2* gene [18]. The associations between *ABCG2* polymorphism and DTG pharmacokinetics were found [10,24]. In addition, the pregnane X receptor (PXR; *NR1I2*) regulates expression of CYP3A4 and UGT1A1. Evidence indicates that the *NR1I2* 63396 TT genotype significantly increases DTG maximum concentrations (C_max_) and the 24 h area under the concentration–time curve (AUC_0–24_) [10]. Moreover, individuals having both UGT1A1*28 and *NR1I2* 63396 TT, or the combination of *ABCG2* 421 AA and *NR1I2* 63396 TT, further amplified DTG exposure, with C_max_ increases of 43–47% and AUC_0–24_ increases of 39–79% compared to those with single genetic variations [10]. The frequency of these gene variations and their impact on DTG exposures may differ by ethnicity, with insufficient data available for the Thai population. This study aimed to investigate the impact of *UGT1A1*, *ABCG2*, and *NR1I2* polymorphisms on the pharmacokinetics of DTG. The findings of this study will enhance the understanding of the factors that affect DTG pharmacokinetics in Thai PLWH.

## 2. Materials and Methods

This cross-sectional analysis was a sub-study of 2 clinical trials performed in Thai PLWH at the HIV Netherlands Australia Thailand Collaboration (HIV-NAT), Thai Red Cross AIDS and Infectious Diseases Research Centre in Bangkok, Thailand (ClinicalTrials.gov registration no. NCT03727152, 1 November 2018 and NCT03785106, 24 December 2018). The NCT03727152 study was a phase III, open-label, single-arm study of virologically suppressed HIV-infected individuals over 18 years. Participants receiving the protease inhibitor/ritonavir were switched to a generic single-tablet regimen of TAF/FTC/DTG. All individuals were orally administered 50 mg of DTG. For the pharmacokinetic sub-study, intensive blood samples were collected at pre-dose and 1, 2, 4, 6, 8, 10, 12, and 24 h post-dose at weeks 24 and 48. The NCT03785106 study was a multicenter, randomized, open-label, phase III clinical trial that compared a 4-week daily isoniazid/rifapentine regimen (1HP) to a 12-weekly isoniazid/rifapentine regimen (3HP) for the treatment of latent tuberculosis infection (LTBI) in PLWH without active TB. Participants were on ART with DTG 50 mg plus TAF or TDF combined with FTC or 3TC. For the PK sub-study, blood samples were collected at pre-dose and 1, 2, 4, 6, 8, 10, 12, and 24 h post-dose during weeks 0 and 4, or between weeks 4 and 12, depending upon the LTBI treatment regimen. Only PK data collected at week 0, prior to LTBI therapy initiation, were included in this analysis. Demographic and laboratory data, including age, sex, body weight, serum creatinine, and liver function (ALT), were recorded. The PK sub-study was approved by the Institutional Review Board of the Faculty of Medicine, Chulalongkorn University, Bangkok, Thailand, and the Institutional Review Board Committee on Human Research at the Faculty of Pharmacy, Chiang Mai University, Chiang Mai, Thailand.

### 2.1. Determination of Dolutegravir Plasma Concentration

Blood samples were processed within 1 h after collection to attain plasma and were stored at −20 °C at the HIV-NAT laboratory. Stored samples designated for DTG concentration analysis were subsequently shipped on dry ice to the Center for Personalized Precision Medicine of Tuberculosis, Inje University College of Medicine, Busan, Republic of Korea. DTG plasma concentrations were determined using validated LC-MS/MS. The intraday and interday precisions were below 15% and accuracy was between 83.5% and 118.4%. The lower limit of quantification was 0.1 mg/L.

### 2.2. Pharmacokinetics

Pharmacokinetic parameters, including area under the 24 h concentration–time curve (AUC_0–24_, h×mg/L), maximum concentration (C_max_, mg/L), 24 h trough concentration (C_trough_, mg/L), and time to reach the maximum concentration (T_max_, h) were calculated using a non-compartmental model by Phoenix WinNonlin (version 8.5.2.4, Certara USA, Inc., Princeton, NJ, USA).

### 2.3. Genotyping Assay

Genomic DNA was extracted from peripheral blood mononuclear cells using the QIAamp DNA blood mini kit (Qiagen, Hilden, Germany) according to the manufacturer’s protocol. Extracted DNA was quantified by NanoDrop (Thermo Fisher Scientific, Wilmington, DE, USA). The promoter region of UGT1A1 was amplified by polymerase chain reaction (PCR) with a forward primer (5′-AAA TTC CAG CCA GTT CAA CTG TTG TT-3′) and a reverse primer (5′-CTG CTG GAT GGC CCC AAG-3′). The PCR conditions were as follows: 34 cycles of denaturation at 94 °C for 45 s, annealing at 62 °C for 45 s, and extension at 72 °C for 60 s with the initial denaturation at 94 °C for 2 min and the final extension at 72 °C for 1 min. The amplified DNA fragments were sequenced by Sanger DNA sequencing. The variants of UGT1A1*28 (TA7), *36 (TA5), and *37 (TA8) were determined by Sequence Scanner Software v2.0 (Thermo Fisher Scientific, USA).

Three single nucleotide polymorphisms, UGT1A1*6 211 G>A (rs4148323), *ABCG2* 421 C>A (rs2231142), and *NR1I2* 63396 C>T (rs2472677) were determined by TaqMan allelic discrimination assay on a QuantStudio7 Flex Real-Time PCR System (Applied Biosystems Inc., Waltham, MA, USA), according to the manufacturer’s standard protocol. The real-time PCR conditions were as follows: 95 °C for 10 min, followed by 40 cycles at 95 °C for 15 s and 60 °C for 1 min.

### 2.4. Statistical Analyses

Statistical analysis was performed using STATA version 14.0 (StataCorp LP, Texas, USA) software. The chi-squared test was used to compare the observed and predicted genotype frequencies in accordance with Hardy–Weinberg equilibrium. The genetic polymorphisms of *UGT1A1* were categorized to designate *UGT1A1* phenotypes in accordance with the CPIC recommendations: extensive metabolizer (*1/*1, *1/*36, *36/*36), intermediate metabolizer (*1/*28, *1/*37, *1/*6, *36/*28, *36/*37), and poor metabolizer (*28/*28, *28/*37, *37/*37, *6/*6) [19].

The normality test carried out by the Shapiro–Wilk test was applied to all pharmacokinetic parameters, with *p* < 0.05 being statistically significant. The pharmacokinetic parameters were natural logarithm (ln) transformed if the normality was not met. The statistical analyses were performed following the natural logarithm (ln) transformation of C_max_ and AUC_0–24_. The pharmacokinetic parameters of DTG (T_max_, C_max_, C_trough_, and AUC_0–24_) were compared among genetic variants of *ABCG2* and *NR1I2*, as well as *UGT1A1* phenotypes, using one-way ANOVA followed by post hoc analysis. A linear regression analysis was employed to evaluate the potential association between dolutegravir C_trough_ and ln AUC_0–24_ and demographic data, including age, sex, body weight, serum creatinine concentration, liver function (ALT), genetic polymorphisms of *ABCG2*, genetic polymorphisms of *NR1I2*, and genetic polymorphisms of *UGT1A1*. Independent variables with a *p*-value of <0.1 in the univariable analysis were included into a multivariable analysis model using the stepwise method. A *p*-value of <0.05 was considered statistically significant for multivariable analysis.

## 3. Results

A total of 104 PLWH were included in the analysis, with baseline characteristics summarized in Table 1. All samples were successfully genotyped for all genetic variations, including UGT1A1*6 211 G>A (rs4148323), *ABCG2* 421 C>A (rs2231142), *NR1I2* 63396 C>T (rs2472677), and *UGT1A1* polymorphisms of the promoter region. The frequencies of genetic polymorphisms for *ABCG2*, *NR1I2*, and *UGT1A1* are presented in Table 2, all of which adhere to Hardy–Weinberg equilibrium (*p*-value > 0.05). The frequencies of *UGT1A1* phenotypes based on CPIC guidelines are shown in Table 3.

### Relationship Between DTG Pharmacokinetic Parameters and Genetic Polymorphisms

No significant association was observed between the *ABCG2* 421 C>A genetic polymorphism and DTG pharmacokinetic parameters. Participants with the *NR1I2* 63396 CC genotype had a significantly higher T_max_ compared with those carrying the *NR1I2* 63396 CT genotype (*p* = 0.014).

One-way ANOVA analysis revealed significant differences in ln C_max_, ln AUC_0–24_, and C_trough_ across *UGT1A1* phenotypes (*p*-values = 0.008, 0.020, and 0.043, respectively). PLWH identified as poor metabolizers exhibited significant increases in ln C_max_, ln AUC_0–24_, and C_trough_ by 34.43%, 34.96%, and 39.20%, respectively, in comparison to extensive metabolizers (Table 4); however, the post hoc analysis confirmed significantly lower C_max_ in extensive metabolizers compared to poor metabolizers (*p*-value = 0.006).

Table 5 shows the impact of genetic and non-genetic factors on DTG ln AUC_0–24_ and C_trough_. In multivariable analyses, both body weight and *UGT1A1* poor metabolizer were independently associated with DTG ln AUC_0–24_ and C_trough_. After accounting for the impact of *UGT1A1* genotypes, each 10 kg increase in body weight was associated with a 1.94% reduction in ln AUC_0–24_ and a 5.91% decrease in C_trough_. Likewise, after adjusting for body weight, PLWH with the *UGT1A1* poor metabolizer demonstrated increases in ln AUC_0–24_ and C_trough_ of 5.18% and 20.59%, respectively.

## 4. Discussion

DTG is a cornerstone of first-line HIV treatment regimens globally due to its high efficacy and favorable safety profile in initial clinical trials. However, transition into real-world clinical practice has unveiled a significant challenge characterized by a high degree of inter-individual DTG pharmacokinetic variability, with reported coefficients of variation (%CV) for exposure parameters reaching as high as 85% [10]. This variability has clinical significance, as elevated DTG exposure has been associated with NP-AEs [2,6,7,9], a common reason for treatment discontinuation [6,7,25,26,27]. Identifying genetic and non-genetic determinants of DTG concentrations is therefore essential, particularly among Thai PLWH.

DTG is primarily metabolized by UGT1A1 [18]. Therefore, genetic polymorphisms of this enzyme may be associated with variations in the pharmacokinetics of DTG. In this study, *UGT1A1* poor metabolizers demonstrated markedly higher DTG exposure, with increases of 34.4% in C_max_, 35.0% in AUC_0–24_, and 39.2% in C_trough_ compared with extensive metabolizers. The geometric mean C_max_ was significantly higher in poor metabolizers (5.70 mg/L) than in extensive metabolizers (4.24 mg/L, *p* = 0.006), consistent with impaired drug clearance in poor metabolizers. These associations persisted after adjusting for the effect of body weight in multivariate analyses: an increase of 5.18% in ln AUC_0–24_ (*p* = 0.018) and a 20.59% increase in C_trough_ (*p* = 0.028) were observed in participants with a UGT1A1 poor metabolizer phenotype. Our results align with prior research by Chen et al., which reported a 46% increase in AUC and a 32% increase in C_max_ among poor metabolizers [28]. Findings from a study by Elliot et al., albeit lacking phenotypic categorization, indicated that the UGT1A1*28 poor metabolizer genotype was independently correlated with higher dolutegravir log_10_ AUC_0–24_. Furthermore, the combination of UGT1A1*28 and UGT1A1*6 resulted in a 36% increase in AUC_0–24_ and a 44% increase in C_trough_ [10]. Yagura et al. discovered that the median DTG C_trough_ was approximately 1.7 times higher in individuals carrying UGT1A1*6 homozygous compared to those carrying both normal alleles. The median C_trough_ was observed to be higher in UGT1A1*28 heterozygous individuals, but not in those with the homozygous genotype, which the authors attributed to insufficient statistical power [23]. This evidence highlights the significance of *UGT1A1* polymorphisms in the pharmacokinetics of DTG. A notable contribution of this study is the characterization of allele frequency in a Thai population, which provides crucial insights into the ethnic-specific distribution of relevant alleles. The allele frequency for UGT1A1*6 (211G>A) was 7.21% and for UGT1A1*28 was 20.67% in our cohort. This is consistent with known distribution: UGT1A1*6 is characteristically more prevalent in East and Southeast Asian populations, while UGT1A1*28 is more common among Caucasians and Africans [29,30]. This highlights the necessity of investigating UGT1A1*6 in Asian cohorts, as its impact may be underappreciated in research focused predominantly on Caucasian populations where this allele is rare. Thus, the use of the CPIC-defined phenotype classification, which integrates information from multiple alleles into a clinically translatable score should be a powerful strategy to investigate the impact of *UGT1A1* polymorphisms on the drug’s pharmacokinetics.

Our findings highlighting the pivotal role of UGT1A1 in DTG pharmacokinetics have significant clinical implications for the second-generation INSTI class. Similarly to DTG, raltegravir and cabotegravir are predominantly metabolized by UGT1A1. Research indicates that reduced-function *UGT1A1* alleles (UGT1A1*6, UGT1A1*28, UGT1A1*37) are correlated with a modest increase in exposure to raltegravir and cabotegravir [20,22]. Conversely, bictegravir is metabolized by both UGT1A1 and CYP3A4. Despite the absence of evidence on the impact of *UGT1A1* polymorphisms on bictegravir, prior research indicated that the inhibition of UGT1A1 alone is unlikely to provide a significant clinical effect. However, concurrent inhibition of both CYP3A4 and UGT1A1 results in a marked increase in bictegravir exposure, with up to a 315% increase in AUC extrapolated to infinity (AUC_0-inf_) [31]. This data indicates a common pharmacogenetic sensitivity within the INSTI class, particularly with DTG, raltegravir, and cabotegravir, although the clinical relevance of this genetic variation may vary among those drugs. Alongside genetic factors, this study identified body weight as an independent non-genetic determinant of DTG pharmacokinetics. The multivariable analysis demonstrated a clear inverse relationship: for every 10 kg increase in body weight, the ln AUC_0–24_ decreased by 1.94% and the C_trough_ decreased by 5.91%. Our results are in good accordance with previous population PK analyses of DTG, which have also identified body weight as a significant covariate influencing DTG apparent clearance [11,12]. Consequently, an individual with a low body weight who is a UGT1A1 poor metabolizer may encounter substantially elevated DTG exposure, thereby increasing the likelihood of NP-AEs or other DTG-related toxicity.

This study found no statistically significant association between the *ABCG2* 421C>A (rs2231142) polymorphism and DTG pharmacokinetic parameters. Notably, prior studies have reported inconsistent results. Adults carrying the variant A allele, particularly the homozygous AA variant, were associated with increased DTG exposure. Elliot RE et al. and Tsuchiya K et al. both reported an increase in DTG C_max_ in adult PLWH with the AA genotype [10,24]. In contrast, a study in children found the opposite effect [32]. Our findings align with Zhu J et al., who demonstrated that *ABCG2* deficiency does not affect the pharmacokinetics of DTG in mice [33]. These discrepancies may reflect population-specific effects, differences in study design, or limited power. In our cohort, only 10 participants (9.62%) were homozygous for the AA variant, reducing the ability to detect a modest effect of this genetic polymorphism. Therefore, the impact of the *ABCG2* 421C>A genotype across diverse populations needs to be further investigated.

We observed that individuals with the *NR1I2* 63396 CC genotype had a significantly longer time to reach T_max_ than those with the heterozygous CT genotype (2.67 vs. 1.68 h, *p* = 0.014). However, in the absence of change in C_max_ or AUC_0–24_, this isolated difference is unlikely to be of clinical relevance.

Our study has several limitations. First, *UGT1A1*, the primary enzyme metabolizing both DTG and bilirubin, was implicated, as bilirubin levels influence DTG pharmacokinetics through competitive metabolism [11,16]. However, bilirubin data were unavailable in our cohort, and the influence of bilirubin levels on the DTG pharmacokinetics was not examined. Second, other potentially relevant genetic polymorphisms, such as *CYP3A4*, were not investigated. Given the minor contribution of CYP3A4 to DTG metabolism [18], variations in *CYP3A4* are unlikely to have a major effect on DTG pharmacokinetics. Third, the low frequency of the homozygous variant genotype for *ABCG2* 421 AA rendered the study insufficiently powered to identify modest genetic effects, hence directly elucidating certain null findings. Lastly, due to the absence of pharmacodynamic outcome data, we are unable to directly associate the observed DTG pharmacokinetic parameters with clinical outcomes in this study. This outcome signifies a crucial trajectory for forthcoming study to clinically translate these genetic and non-genetic factors into individualized HIV therapy strategies. Future study validating these correlations in larger cohorts should be conducted. Additionally, the optimization of DTG dose to attain maximum efficacy and minimum toxicity warrants investigation.

## 5. Conclusions

In conclusion, this study provides evidence that body weight and *UGT1A1* poor metabolizer significantly impact DTG pharmacokinetics. Individuals with a *UGT1A1* poor metabolizer genotype, particularly those with a lower body weight, could be predisposed to supratherapeutic DTG concentrations, which may increase susceptibility to concentration-dependent adverse events, such as the neuropsychiatric side effects that have been a concern in real-world clinical practice.

## Figures and Tables

**Table 1:** Demographic characteristics of study participants.

Characteristic	Value
Sex, frequency (%)	
Male	53 (51.0)
Female	51 (49.0)
Body weight (kg), median ± IQR (min–max)	62.3 ± 17.3 (41.3–115.1)
Age (years), median ± IQR (min–max)	30.5 ± 27.0 (18.0–68.0)
Serum creatinine (mg/dL), median ± IQR (min–max)	0.8 ± 0.3 (0.5–1.5)
ALT (U/L), median ± IQR (min–max)	31.0 ± 17.0 (13.0–76.0)
Height (m), median ± IQR (min–max)	1.6 ± 0.1 (1.5–1.9)
BMI (kg/m^2^), median ± IQR (min–max)	22.9 ± 5.6 (19.0–36.0)

ALT, Alanine Aminotransferase; BMI, Body Mass Index.

**Table 2:** Frequencies of genetic polymorphisms of *ABCG2, NR1I2* and *UGT1A1* (N = 104).

Genetic Polymorphisms	Genotype			Allele	
	Genotype	No. of PLWH	% of PLWH	Allele	Frequency (%)
*ABCG2* 421C>A (rs2231142)	CC	57	54.8	C	72.6
	CA	37	35.6	A	27.4
	AA	10	9.6		
*NR1I2* 63396C>T (rs2472677)	CC	15	14.4	C	38.0
	CT	49	47.1	T	62.0
	TT	40	38.5		
UGT1A1*6 221G>A (rs4148323)	GG	89	85.6	G	92.8
	GA	15	14.4	A	7.2
	AA	0	0		
*UGT1A1* (TA)_n_ repeats	TA6/TA6	66	63.5	TA6	78.8
	TA6/TA7	32	30.8	TA7	20.7
	TA7/TA7	5	4.8	TA8	0.5
	TA7/TA8	1	0.9		

**Table 3:** Frequencies of *UGT1A1* phenotypes.

*UGT1A1* Phenotypes	Genotype	No. of PLWH (%)
Extensive metabolizer	An individual carrying two reference function (*1) and/or increased function alleles (*36)	55 (52.9)
Intermediate metabolizer	An individual carrying one reference (*1) and one decreased function allele (*6, *28, *37)	39 (37.5)
Poor metabolizer	An individual carrying two decreased functions alleles (*6, *28, *37)	10 (9.6)

**Table 4:** Association between dolutegravir pharmacokinetic parameters and *ABCG2*, *NR1I2* genotypes, and *UGT1A1* phenotypes.

Genetic Polymorphism	Pharmacokinetic Parameter	Genotype	Geometric Mean ^a^	%CV	*p*-Value ^#^	Post Hoc Analysis	*p*-Value ^&^
*ABCG2* 421 C>A (rs2231142)	T_max_ (hour)	CC	2.05	1.1	0.503		
		CA	1.87	1.3			
		AA	2.35	1.3			
	C_max_ (mg/L)	CC	4.55	59.4	0.558		
		CA	4.29	27.3			
		AA	4.26	19.1			
	AUC_0–24_ (mg×hour/L)	CC	59.39	34.6	0.598		
		CA	55.42	31.6			
		AA	57.17	27.4			
	C_trough_ (mg/L)	CC	1.38	0.6	0.651		
		CA	1.28	0.6			
		AA	1.28	0.5			
*NR1I2* 63396 C>T (rs2472677)	T_max_ (hour)	CC	2.67	1.3	0.009 *	CC-CT	0.014 **
		CT	1.68	0.9		CC-TT	0.508
		TT	2.18	1.4		CT-TT	0.132
	C_max_ (mg/L)	CC	4.40	36.0	0.399		
		CT	4.41	27.5			
		TT	4.44	28.1			
	AUC_0–24_ (mg×hour/L)	CC	56.89	46.3	0.939		
		CT	57.41	29.6			
		TT	58.45	31.8			
	C_trough_ (mg/L)	CC	1.27	0.7	0.844		
		CT	1.32	0.5			
		TT	1.37	0.6			
*UGT1A1* phenotypes	T_max_ (hour)	Extensive metabolizer	2.10	1.2	0.732		
		Intermediate metabolizer	1.90	1.2			
		Poor metabolizer	2.03	1.2			
	C_max_ (mg/L)	Extensive metabolizer	4.24	27.4	0.008 *	Extensive-Intermediate	1.000
		Intermediate metabolizer	4.40	28.4		Extensive-poor	0.006 **
		Poor metabolizer	5.70	25.6		Intermediate-poor	0.025
	AUC_0–24_ (mg×hour/L)	Extensive metabolizer	55.98	30.8	0.020 *	Extensive-Intermediate	1.000
		Intermediate metabolizer	56.28	33.3		Extensive-poor	0.019
		Poor metabolizer	75.55	31.1		Intermediate-poor	0.030
	C_trough_ (mg/L)	Extensive metabolizer	1.25	0.5	0.043 *	Extensive-Intermediate	1.000
		Intermediate metabolizer	1.34	0.6		Extensive-poor	0.037
		Poor metabolizer	1.74	0.7		Intermediate-poor	0.138

^#^ *p*-value was calculated using one-way ANOVA; * *p* < 0.05; ^&^ *p*-value was calculated post hoc analysis using Bonferroni correction; ** *p* < 0.0167; ^a^ T_max_ and C_trough_ were calculated using arithmetic mean and SD. T_max_, Time to maximum concentration; C_max_, maximum concentration; C_trough_, 24 h trough concentration; AUC_0–24_, area under the 24 h concentration–time curve.

**Table 5:** Univariable and Multivariable analyses of genetic and nongenetic factors for dolutegravir.

	Univariable Analysis			Multivariable Analysis ^b^		
Factor	Coefficient	95% CI	*p*-Value ^a^	Coefficient	95% CI	*p*-Value ^a^
**ln AUC_0–24_ (mg×hour/L)**						
Female	0.073	−0.052 to 0.198	0.250			
Age (year)	−0.002	−0.006 to 0.002	0.335			
Body weight (kg)	−0.010	−0.013 to −0.005	<0.001 *	−0.009	−0.014 to −0.005	<0.001 **
Serum creatinine (mg/dL)	0.036	−0.290 to 0.362	0.826			
ALT (U/L)	−0.005	−0.011 to 0.001	0.125			
*UGT1A1* phenotypes						
Extensive metabolizer		Reference			Reference	
Intermediate metabolizer	0.008	−0.122 to 0.138	0.901			
Poor metabolizer	0.299	0.086 to 0.513	0.006 *	0.240	0.041 to 0.438	0.018 **
*ABCG2* 421 C>A						
CC		Reference				
CA	−0.069	−0.205 to 0.066	0.314			
AA	−0.038	−0.258 to 0.182	0.734			
*NR1I2* 63396 C>T						
CC		Reference				
CT	0.012	−0.178 to 0.203	0.897			
TT	0.031	−0.164 to 0.226	0.752			
**C_trough_ (mg/L)**						
Female	0.026	−0.198 to 0.250	0.816			
Age (year)	−0.0001	−0.008 to 0.008	0.980			
Body weight (kg)	−0.013	−0.021 to −0.005	0.002 *	−0.012	−0.020 to −0.004	0.005 **
Serum creatinine (mg/dL)	0.311	−0.267 to 0.888	0.288			
ALT (U/L)	−0.006	−0.017 to 0.005	0.259			
*UGT1A1* phenotypes						
Extensive metabolizer		Reference			Reference	
Intermediate metabolizer	0.090	−0.143 to 0.323	0.444			
Poor metabolizer	0.492	0.109 to 0.874	0.012 *	0.418	0.045 to 0.790	0.028 **
*ABCG2* 421 C>A						
CC		Reference				
CA	−0.106	−0.347 to 0.136	0.387			
AA	−0.105	−0.497 to 0.287	0.597			
*NR1I2* 63396 C>T						
CC		Reference				
CT	0.047	−0.291 to 0.386	0.781			
TT	0.096	−0.251 to 0.443	0.584			

^a^ Linear regression analysis; *, *p* < 0.1; **, *p* < 0.05; ^b^ Factors with *p*-value of <0.1 in the univariable analysis were entered into the multivariable analysis.

## Data Availability

The data that support the findings of this study are available on request from the corresponding author.

## Abbreviations

The following abbreviations are used in this manuscript:

DTG	Dolutegravir
INSTI	Integrase strand inhibitor
PLWH	People living with HIV
TAF	Tenofovir alafenamide
TDF	Tenofovir disoproxil fumarate
3TC	Lamivudine
FTC	Emtricitabine
IC90	in vitro protein-adjusted 90% inhibitory concentration
C_trough_	Trough concentration
NP-AEs	Neuropsychiatric adverse events
%CV	Percent coefficient of variation
UGT1A1	Uridine diphosphate glucuronosyltransferase family 1 member A1
CYP	Cytochrome P450
BCRP	Breast cancer resistance protein
ABCG2	ATP binding cassette subfamily G member 2
PXR	Pregnane X receptor
NR1I2	Nuclear receptor subfamily 1 group I member 2
C_max_	Maximum concentration
AUC_0–24_	Area under the concentration–time curve from 0–24 h post-dose
HIV-NAT	The HIV Netherland Australia Thailand collaboration
LTBI	Latent tuberculosis infection
T_max_	Time to reach the maximum concentration
ln	Natural logarithm
IQR	Interquartile range
Min	Minimum value
Max	Maximum value
Kg	Kilogram
ALT	Alanine aminotransferase
BMI	Body mass index
N	Number of participants
No.	Number of participants
CI	Confidence interval
